# Levels of microRNA-181b and plasminogen activator inhibitor-1 are associated with hypertensive disorders complicating pregnancy

**DOI:** 10.3892/etm.2014.1946

**Published:** 2014-09-02

**Authors:** YAN-SHAN CHEN, LING SHEN, RUI-QIN MAI, YING WANG

**Affiliations:** 1 Department of Gynecology and Obstetrics, The First Affiliated Hospital of Shantou University Medical College, Shantou, Guangdong 515041, P.R. China; 2 Department of Clinical Laboratory, The First Affiliated Hospital of Shantou University Medical College, Shantou, Guangdong 515041, P.R. China

**Keywords:** hypertensive disorders complicating pregnancy, microRNA-181b, plasminogen activator inhibitor-1

## Abstract

The aim of the present study was to explore the association between the expression of microRNA (miRNA)-181b and plasminogen activator inhibitor-1 (PAI-1) in the placental tissue of pregnant females with a hypertensive disorder complicating pregnancy (HDCP). Placental tissue samples were obtained from 48 patients with HDCP and 40 females with a normal pregnancy. The levels of miRNA-181b and PAI-1 mRNA were determined by the reverse transcription-quantitative polymerase chain reaction (RT-qPCR). The expression of PAI-1 protein was analyzed by western blotting. Vascular smooth muscle cells (VSMCs) were transfected with the pEGP-miRNA-181b plasmid using Lipofectamine^®^ 2000. Transfection efficiency was confirmed by immunohistochemical analysis. The levels of miRNA-181b in the placental tissue of patients with HDCP were lower than those in the control group, whereas the levels of PAI-1 mRNA in the placental tissue of patients with HDCP were higher than those in the control group. The expression of the PAI-1 protein in the HDCP group was higher than that in the control group. Following transfection of VSMCs with plasmid pGCMV/EGFP/miRNA-181b, the levels of PAI-1 mRNA were reduced while the levels of miRNA-181 were upregulated. Furthermore, the expression levels of PAI-1 protein were lower than those in the control group. The levels of miRNA-181b and PAI-1 mRNA were strongly associated with HDCP. Thus, miRNA-181b may play an important role in the regulation of PAI-1. PAI-1 and miRNA-181b may be novel biomarkers to be used in HDCP therapy.

## Introduction

Hypertensive disorder complicating pregnancy (HDCP) is a common clinical obstetric complication with an incidence rate of 5% in China (1). Due to a lack of effective means of prevention and treatment, HDCP threatens the lives of pregnant females and their fetuses. The clinical manifestations of HDCP are mainly hypertension, proteinuria and edema, as well as convulsion, coma and heart or kidney failure when aggravated (1). Studies on molecular biomarkers for the early stages of HDCP development are important for the prevention and treatment of HDCP. microRNA (miRNA)-181b, a member of the miRNA-181 family, has been revealed to be involved in the proliferation, invasion and apoptosis of tumor cells that are associated with tumor invasiveness (2,3). Plasminogen activator inhibitor-1 (PAI-1), a novel target for the clinical treatment of cardiovascular disease (4), inhibits fibrinolysis, induces barriers for extracellular matrix degradation, impacts the invasiveness of cells (5) and plays an important role in vascular structural and functional changes (6).

Previous studies on the pathological mechanisms of HDCP have demonstrated that the placenta plays an important role in the occurrence and development of HDCP; the mechanism is associated with the reduced invasiveness of trophoblastic cells and pathological changes in small branches of the main uterine artery (7,8). Furthermore, it is hypothesized that the shallow invasion of trophoblastic cells into the uterus, dysfunctional physiological recovery of the spiral arterioles and placental ischemia and hypoxia are associated with HDCP (9). Studies have indicated that miRNA-181 has an effect on the proliferation of cells, the regulation of angiogenesis, the conditioning of the immune system (10), tumor invasion and apoptosis (11). Furthermore, miRNA-181 has been shown to exhibit clinical value by regulating Bcl-2, OPN and K-ras genes (12,13). Notably, miRNA-181b has been demonstrated to promote the proliferation and invasiveness of multiple tumor cells and is closely associated with tumor apoptosis (14). In addition, a previous study revealed that miRNA-181a promotes the invasiveness of trophoblastic cells and is highly expressed in the placental tissue of patients with HDCP (15). However, the effect of miRNA-181b on the occurrence and development of HDCP has not, to the best of our knowledge, been previously studied.

PAI-1 is mainly expressed in vascular endothelial cells and vascular smooth muscle cells (VSMCs), and plays an important role in the hypertrophy, proliferation and remodeling of the VSMCs (16). Bioinformatic studies have indicated that the 3′-untranslated region of PAI-1 mRNA is complementary to miRNA-181b (17,18). Therefore, we hypothesized that miRNA-181b may be capable of regulating the expression of PAI-1, which affects vascular structural remodeling and function, thereby promoting the occurrence and development of HDCP. In the present study, the mechanisms of action of miRNA-181b and PAI-1 in the occurrence and development of HDCP were investigated.

## Materials and methods

### Subjects

Placental tissue was collected from 48 puerperae with HDCP (HDCP group) and 40 puerperae with normal deliveries (normal control group) between October 2011 and October 2013. In the HDCP group, 28 females had gestational hypertension (high blood pressure following 20 weeks of pregnancy; no proteinuria), 15 females had mild pre-eclampsia (systolic pressure ≥140 mmHg or diastolic pressure ≥90 mmHg; 24 h proteinuria ≥300 mg) and five females had severe pre-eclampsia (systolic pressure ≥160 mmHg or diastolic pressure ≥100 mmHg; 24 h proteinuria ≥2 g). The ages of the females in the HDCP group ranged between 24 and 34 years, with an average age of 28.5 years and a median age of 27 years. The ages of the females in the normal control group ranged between 25 and 30 years, with an average age of 26.5 years and a median age of 25 years. Written informed consent was obtained from all participants and all experiments were approved by the Ethics Committee of Shantou University (Shantou, China).

### Reagents

VSMCs were purchased from the Cell Bank of Chinese Academy of Sciences (Shanghai, China). Plasmids for pGCMV/EGFP/miRNA-181b and the negative control were constructed by Shanghai GenePharma Co., Ltd. (Shanghai, China). Rabbit anti-human PAI-1 polyclonal antibody was purchased from Abcam (Cambridge, MA, USA). An RNA extraction kit (spin column) was purchased from Sangon Biotech Co., Ltd. (Shanghai, China). A reverse transcription kit was purchased from Chengdu Boruike Biotech Co., Ltd. (Chengdu, China). First-strand miRNA/cDNA synthesis kits were purchased from Beijing CoWin Biotech Co., Ltd. (Beijing, China). SYBR Green reverse transcription-quantitative polymerase chain reaction (RT-qPCR) reagents were purchased from Kapa Biosystems (Boston, MA, USA). Lipofectamine^®^ 2000 was purchased from Invitrogen Life Technologies (Carlsbad, CA, USA).

### Total RNA extraction

Total RNA from the placental tissue and VSMCs was extracted using the total RNA extraction kit in accordance with the manufacturer’s instructions (Sangon Biotech Co., Ltd.). The quality of the extracted RNA was confirmed by RNA molecular electrophoresis and a NanoDrop ND-1000 UV-Vis spectrophotometer (NanoDrop Technologies, Inc., Wilmington, DE, USA) at an optical density ratio of 260/280 nm.

### RT

Total RNA (1 μg) was used for RT. RNA template (5 μl), *Escherichia coli* poly(A) polymerase (0.4 μl), poly(A) polymerase B buffer (2 μl), adenosine triphosphate solution (2 μl) and RNase-free double distilled water (10.6 μl) were added into a pre-cooled RNase-free Eppendorf tube and incubated at 37°C for 60 min prior to poly(A) modification. The mixture (4 μl) was subsequently added to the RT reaction mixture (20 μl) containing RT primer (2 μl), RT buffer (4 μl), deoxynucleotide triphosphate mix (1 μl), TUREscriptH-RTase (0.9 μl) and RNase-free H_2_O (8.9 μl). The RT reaction was maintained at 42°C for 50 min and heated to 70°C for 15 min prior to termination. The cDNA was stored at −20°C.

### Western blot analysis

Placental tissue (100 mg) was obtained from individuals in the HDCP and normal control groups and ground into a powder in liquid nitrogen. Radioimmunoprecipitation assay lysis buffer and protease inhibitors were added with thorough mixing prior to incubation at 4°C overnight. The following day, the samples were centrifuged at 15,680 × g at 4°C for 10 min and the supernatants were stored in aliquots. Prior to electrophoresis, sample loading buffer (2X) was mixed thoroughly with an aliquot of the sample before denaturation for 5 min in a boiling water bath. The mixture (10 μl) was loaded onto the gel for sodium dodecyl sulfate polyacrylamide gel electrophoresis at a constant 80 V and electrically transferred onto a polyvinylidene difluoride membrane at constant 200 mA in an ice bath for 2 h. The membrane was blocked by skimmed milk (50 g/l) for 1 h at room temperature. Primary antibodies against PAI-1 (1:1,000; Abcam) and β-actin (1:5,000; Abcam) were added prior to incubation with agitation overnight at 4°C. Following rinsing with phosphate-buffered saline (PBS) with Tween 20 three times for 10 min, secondary antibodies labeled with horseradish peroxidase (goat anti-mouse, 1:5,000; goat anti-rabbit, 1:2,000; Abcam) were added prior to incubation at room temperature for 1 h. This was followed by rinsing with PBS with Tween 20 three times for 10 min. The immunoreactive bands were visualized by electrochemiluminescence.

### RT-qPCR

U6 and GAPDH were used as internal controls for the quantification of miRNA-181b and PAI-1, respectively. The miRNA-181b reaction system was composed of the RT-qPCR mix (10 μl), upstream and downstream primers (0.5 μl each; Invitrogen Life Technologies), cDNA (5 μl) and double distilled H_2_O (13 μl). The amplification conditions were as follows: Initial denaturation at 95°C for 10 min, denaturation at 95°C for 30 sec, annealing at 65°C for 30 sec and extension at 72°C for 1 min, repeated for 40 cycles. The samples were analyzed in triplicate. The PAI-1 reaction system was composed of the RT-qPCR mix (10 μl), upstream and downstream primers (0.5 μl each; Invitrogen Life Technologies), cDNA (1 μl) and double distilled H_2_O (8 μl). The amplification conditions were as follows: Initial denaturation at 95°C for 10 min, denaturation at 95°C for 1 min, annealing at 58°C and 72°C for 30 sec, and extension at 72°C for 1 min, repeated for 40 cycles. The samples were analyzed in triplicate.

### Cell transfection

On the day prior to transfection, VSMCs (1×10^5^) in the logarithmic phase were seeded onto 24-well plates and divided into the normal control, miRNA-181b-VSMC and miRNA negative control groups. The cells were cultured in antibiotic-free, high-glucose Dulbecco’s modified Eagle’s medium (DMEM) and 10% fetal bovine serum (FBS). The cells were transfected when 70% confluency was reached. The pGCMV/EGFP/miRNA-181b plasmid (2 μg) and Lipofectamine 2000 (1 μl) were mixed individually with Opti-MEM^®^ I (50 μl; Invitrogen Life Technologies) in separate Eppendorf tubes and left to stand for 5 min prior to being mixed into one tube. After standing for 20 min at room temperature, the mixture was added into the wells of the culture plates. After 6 h, the medium was replaced with fresh high-glucose DMEM and 10% FBS. After 48 h, green fluorescence was observed under an inverted fluorescence microscope (X51; Olympus Corporation, Tokyo, Japan) for the preliminary analysis of transfection efficiency. After 48 and 72 h of transfection, the cells were analyzed to determine the expression levels of the genes and proteins.

### Statistical analysis

The results were analyzed using SPSS 16.0 software (SPSS, Inc., Chicago, IL, USA). All results are expressed as the mean ± standard deviation. Two groups of mean values were compared using the Student’s t-test. P<0.05 was considered to indicate a statistically significant difference.

## Results

### HDCP reduces the levels of miRNA-181b but increases the levels of PAI-1 mRNA in placental tissue

**Figure 1:**
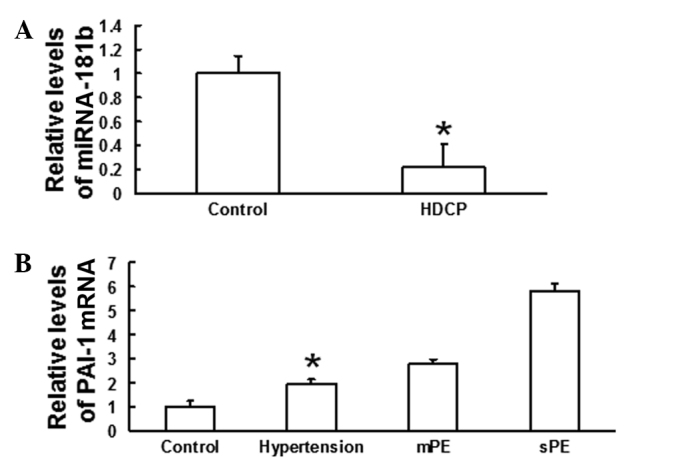
Levels of (A) miRNA-181b and (B) PAI-1 mRNA in the normal control and HDCP groups. The HDCP group was divided into three subgroups of hypertension, mPE and sPE. Data were normalized against those of the normal control group and expressed as the mean ± standard deviation. P<0.05 was considered significantly different. ^*^Significant difference from the control. miRNA-181b, microRNA-181b; PAI-1, plasminogen activator inhibitor-1; HDCP, hypertensive disorder complicating pregnancy; mPE, mild pre-eclampsia; sPE, severe pre-eclampsia.

To determine the levels of miRNA-181b and PAI-1 mRNA in the placental tissue from pregnant females in the HDCP and control groups, RT-qPCR was performed. The results revealed that the level of miRNA-181b in the placental tissue from the HDCP group was significantly lower than that in the placental tissue from the control group (P<0.05; Fig. 1A). By contrast, the level of PAI-1 mRNA in the placental tissue from the HDCP group was higher than that in the placental tissue from the control group. The levels of PAI-1 mRNA in the placental tissue from patients with gestational hypertension, mild pre-eclampsia and severe pre-eclampsia increased 1.98-, 2.79- and 5.8-fold, respectively, compared with those from the control group (Fig. 1B). These data indicated that HDCP reduced the levels of miRNA-181b but increased the levels of PAI-1 mRNA in placental tissue.

### HDCP increases the protein expression of PAI-1

**Figure 2:**
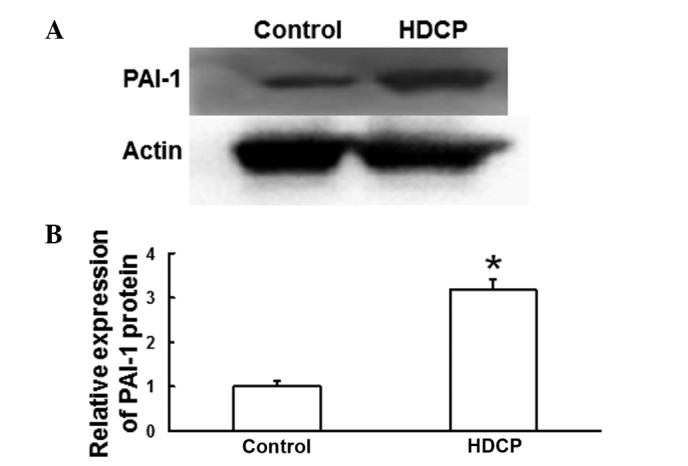
Expression of PAI-1 protein in placental tissue. (A) Western blot analysis of PAI-1 protein expression in the normal control and HDCP groups. (B) Quantification of the relative expression of PAI-1 protein in the normal control and HDCP groups. Data from the HDCP group were normalized against those from the normal control group and expressed as the mean ± standard deviation. P<0.05 was considered significantly different. ^*^Significant difference from the control. PAI-1, plasminogen activator inhibitor-1; HDCP, hypertensive disorder complicating pregnancy.

To measure PAI-1 protein expression, western blot analysis was performed. Western blotting revealed that the expression of PAI-1 protein in placental tissue from the HDCP group was significantly higher than that in placental tissue from the normal control group (P<0.05; Fig. 2). This result suggested that HDCP increased the protein expression of PAI-1.

### Transfection of VSMCs with the pGCMV/EGFP/miRNA-181b plasmid enhances the levels of miRNA-181b and reduces the levels of PAI-1 mRNA in VSMCs

**Figure 3:**
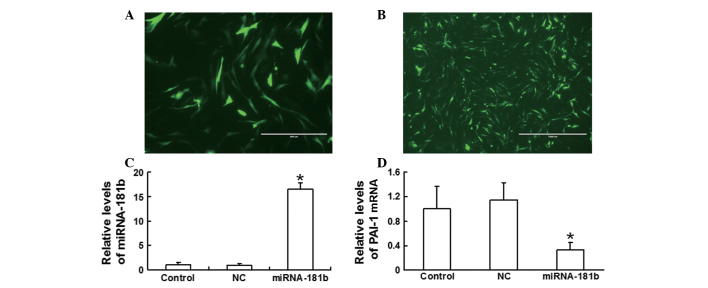
Fluorescence visualization of the VSMCs transfected with (A) an empty vector and (B) miRNA-181b plasmid. (C and D) Levels of (C) miRNA-181b and (D) PAI-1 mRNA in the normal control, NC and miRNA-181b groups following 48 h of transfection, as determined by reverse transcription-quantitative polymerase chain reaction. Data were normalized against those of the normal control group and expressed as the mean ± standard deviation. ^*^P<0.05, vs. normal control. VSMC, vascular smooth muscle cell; miRNA-181b, microRNA-181b; PAI-1, plasminogen activator inhibitor-1; NC, negative control.

To investigate the expression levels of miRNA-181b and PAI-1 in the VSMCs, the pGCMV/EGFP/miRNA-181b plasmid was transfected into the VSMCs and the expression of miRNA-181b was detected using RT-qPCR. Microscopic observation following 48 h (Fig. 3) of transfection revealed that the fluorescence in the transfected VSMCs was distributed evenly (Fig. 3B), with a wider range and a higher density than that in the cells of the negative control group (Fig. 3A). Analysis by RT-qPCR demonstrated that the level of miRNA-181b in the VSMCs following 48 h of transfection was significantly higher than that in normal VSMCs (P<0.05; Fig. 3C). Furthermore, the levels of PAI-1 mRNA in the VSMCs following 48 h of transfection were significantly lower than those in normal VSMCs (P<0.05; Fig. 3D). These data demonstrated that transfection of VSMCs with the pGCMV/EGFP/miRNA-181b plasmid enhanced the levels of miRNA-181b in the VSMCs, in which the levels of PAI-1 mRNA were reduced.

### Transfection of VSMCs with the pGCMV/EGFP/miRNA-181b plasmid reduces PAI-1 protein expression

**Figure 4:**
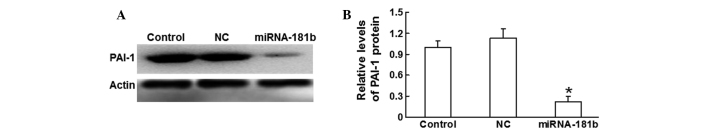
Expression levels of PAI-1 protein in VSMCs. (A) Western blot analysis of PAI-1 protein expression in the normal control, NC and miRNA-181b (miRNA-181b-VSMC) groups. (B) Quantification of the relative expression of PAI-1 protein in the normal control, NC and miRNA-181b groups. Data were normalized against those of the normal control group and expressed as the mean ± standard deviation. ^*^P<0.05, vs. normal control. VSMC, vascular smooth muscle cell; miRNA-181b, microRNA-181b; PAI-1, plasminogen activator inhibitor-1; NC, negative control.

To determine PAI-1 protein expression in VSMCs transfected with the pGCMV/EGFP/miRNA-181b plasmid, western blotting was used to analyze the extracted proteins from VSMCs following 72 h of transfection. The results revealed that the protein expression of PAI-1 in VSMCs transfected with pGCMV/EGFP/miRNA-181b was significantly lower than that in the normal control and negative control groups (P<0.05; Fig. 4). These data indicated that transfection of VSMCs with the pGCMV/EGFP/miRNA-181b plasmid reduced PAI-1 protein expression.

## Discussion

In the present study, the levels of miRNA-181b in the placental tissue from the 48 patients with HDCP were lower than those in the placental tissue of normal pregnant females. However, the mRNA expression levels of PAI-1 in the placental tissue from patients with HDCP were higher compared with those in the placental tissue of normal pregnant females. In addition, the mRNA expression levels of PAI-1 gradually increased with the development of HDCP at different clinical stages (hypertension, mild pre-eclampsia and severe pre-eclampsia). By contrast, the levels of miRNA-181b were independent of the clinical stages of HDCP (data not shown).

Western blot analysis revealed that the protein expression of PAI-1 in the HDCP group was significantly enhanced compared with that in the control group, indicating that PAI-1 plays an important role in HDCP. By contrast, in hyperglycemia, PAI-1 protein expression has been found to decrease (19). This inconsistency indicates that PAI-1 protein expression may be regulated differently between hyperglycemia and HDCP. The changes in the levels of miRNA-181b in the placental tissue were contrary to the changes in the levels of PAI-1, suggesting that miRNA-181b may be involved in the regulation of PAI-1 expression.

Previous studies have demonstrated that miRNA-181 may regulate embryo implantation, placentation and decidualization via the regulation of the focal adhesion signaling pathway (20,21). These observations indicate that the miR-181 family may play an important role in the development of the embryo and placenta. Therefore, to further investigate the role of miRNA-181b, an miRNA-181b eukaryotic expression model was constructed by transfecting VSMCs with miRNA-181b. The RT-qPCR results revealed that the levels of miRNA-181b increased by 16.5-fold, whereas the mRNA expression levels of PAI-1 were reduced by 67% of the levels prior to transfection. Furthermore, the protein expression level of PAI-1 decreased to 22% of the level prior to transfection. These results demonstrated that PAI-1 expression was reduced when miRNA-181b expression was elevated, indicating that miRNA-181b may be involved in the regulation of PAI-1 expression. However, chromatin immunoprecipitation sequencing is required prior to confirming that PAI-1 is the target gene for miRNA-181b regulation.

In conclusion, miRNA-181b and PAI-1 were shown to be pathologically expressed in the placental tissue of patients with HDCP. Therefore, miRNA-181b plays an important role in the occurrence and development of HDCP, possibly through participating in the regulation of PAI-1, although the precise underling mechanism requires further investigation. Thus, miRNA-181b and PAI-1 have a potential clinical value in the early diagnosis and prevention of HDCP.
